# Supplementary material for: Content-rich biological network constructed by mining PubMed abstracts
Source: BMC Bioinformatics. 2004 Oct 8;5:147. doi: 10.1186/1471-2105-5-147 (PMC528731; doi:10.1186/1471-2105-5-147)
Supplement: Additional File 2 — The original results of the above study (non-essential files are deleted to keep the file size under the limit set by BMC bioinformatics). [file 1471-2105-5-147-S2.bz2 › chilibotAdditionalFile2/dip40/453ID10477718E10464/html/CCL2_CCR5.html]

 


 **CCL2** and **CCR5** 
  
Found 168 abstracts in PubMed, retrieved 40.  
 

 What does Google say? 
 PDF only 
| .edu only 

---

**Interactive relationship** (e.g. stimulation, inhibition, etc)

**Stimulatory relationship**- MIP 1alpha, MIP 1beta, RANTES, MCP  [ **CCL2** ]  2 and MCP  [ **CCL2** ]  4 were able to stimulate increases in intracellular calcium ions via activation of  **CCR5**  whereas MCP  [ **CCL2** ]  3 was without effect.  Ref: 11861332 Br J Pharmacol, 2002
- Also, increased expression of IFN gamma was associated with a significant increase in expression of RANTES and monocyte chemotactic protein 1  [ **CCL2** ] , chemokines that may inhibit HIV 1 infection by blocking viral attachment to CCR2 and  **CCR5**  co receptors.  Ref: 12048358 J Acquir Immune Defic Syndr, 2002
- The roles of  **CCR5**  in excitotoxic injury in  **CCR5**  deficient mice are compensated by increased CCR2 and CCR3 expression, which share the common MCP  [ **CCL2** ]  2 ligand with  **CCR5** .  Ref: 12807425 J Neurochem, 2003
**Neutral relationship**- Furthermore, MCP  [ **CCL2** ]  2 induces a robust, pertussis toxin sensitive calcium flux in primary lymphocytes, and cross desensitization studies indicate that MCP  [ **CCL2** ]  2 acts via  **CCR5** .  Ref: 11930329 J Infect Dis, 2002
- A number of inflammatory CC chemokines, including MIP 1 alpha, MIP 1 beta, RANTES, MCP  [ **CCL2** ]  2, and HCC 1 9 74 act as  **CCR5**  agonists, while MCP  [ **CCL2** ]  3 is a natural antagonist of the receptor.  Ref: 12402506 Receptors Channels, 2002
- **CCR5**  is a G protein coupled receptor for RANTES, MIP 1a, MIP 1b and MCP  [ **CCL2** ]  2 that functions as the front line coreceptor for human immunodeficiency virus type 1 infection.  Ref: 12837756 J Biol Chem< MedlineTA>J Biol Chem, 2002
- The present findings suggest that MCP 1  [ **CCL2** ] , RANTES, and IP 10 may participate in the pathogenesis of mercuric chloride induced tubulointerstitial fibrosis with mononuclear cell infiltration, via CCR2, CCR1 or  **CCR5** , and CXCR3, respectively.  Ref: 12834626 Exp Mol Pathol, 2003
- **CCR5**  is a G protein coupled receptor responding to four natural agonists, the chemokines RANTES regulated on activation normal T cell expressed and secreted, macrophage inflammatory protein MIP 1 alpha, MIP 1 beta, and monocyte chemotactic protein MCP  [ **CCL2** ]  2, and is the main co receptor for the macrophage tropic human immunodeficiency virus strains.  Ref: 12411445 J Biol Chem, 2003
- These data confirm that MCP  [ **CCL2** ]  2 is a ligand for  **CCR5**  on CD4 lymphocytes and can specifically block R5 HIV 1.  Ref: 11930329 J Infect Dis, 2002

**Non-interactive relationship** (e.g. studied together, co-existance, homology, etc.)

- Neuropathogenesis of lentiviral infection in macaques roles of CXCR4 and  **CCR5**  viruses and interleukin 4 in enhancing monocyte chemoattractant protein 1  [ **CCL2** ]  production in macrophages.  Ref: 12213709 Am J Pathol, 2002
- Only CpG ODN exacerbated HAF GN with an increase of glomerular macrophages, which was associated with massive albuminuria and increased renal MCP 1  [ **CCL2** ]   **CCL2** , RANTES CCL5, CCR1, CCR2, and  **CCR5**  mRNA expression.  Ref: 12538732 J Am Soc Nephrol, 2003
- In parallel with down regulated SP and  **CCR5**  mRNA expression, cDNA array assays indicated that CP 9 45 treatment also inhibited IL 8 gene expression, while enhancing expression of fractalkine and monocyte chemotactic protein 3 MCP  [ **CCL2** ]  3.  Ref: 12098517 J Neuroimmunol, 2002
- RESULTS Expression of IP 10, Mig, I TAC, RANTES, CXCR3, and  **CCR5** , but not MCP 1  [ **CCL2** ]  and IL 8, increased significantly in both grade 2 and grade 3 rejection P< ; =0.0096.  Ref: 12544874 Transplantation, 2003
- Two hundred nine liver transplant recipients were genotyped using polymerase chain reaction sequence specific primers for the following polymorphisms RANTES 28, MCP 1  [ **CCL2** ]  2518, and  **CCR5**  59029.  Ref: 12462338 J Clin Immunol, 2002
- Analysis of gene polymorphisms in the regulatory region of MCP 1  [ **CCL2** ] , RANTES, and  **CCR5**  in liver transplant recipients.  Ref: 12462338 J Clin Immunol, 2002
- The expression of interferon IFN gamma inducible protein IP 10, monokine induced by IFN gamma Mig, interferon inducible T cell alpha chemoattractant I TAC, regulated on activation normal T cell expressed and secreted RANTES, monocyte chemotactic protein MCP 1  [ **CCL2** ] , interleukin IL 8, and the receptors CXCR3 and  **CCR5**  were tested using quantitative, real time polymerase chain reaction.  Ref: 12544874 Transplantation, 2003
- CONCLUSIONS This study suggested diagnostic as well as potential functional roles of the chemokine receptor pairs IP10 CXCR3, RANTES  **CCR5** , and MCP1  [ **CCL2** ]  CCR2 in rat models of transplant vasculopathy.  Ref: 12398874 J Heart Lung Transplant, 2002
- The presence of chemokine MCP 1  [ **CCL2** ] , MIP 1alpha, MIP 1beta, IP 10, RANTES positive cells and chemokine receptor  **CCR5** , CXCR3 positive cells in inflamed human gingival tissues.  Ref: 12445801 Cytokine, 2002
- We investigated the effects of a number of naturally occurring chemokines MIP 1alpha, MIP 1beta, RANTES, MCP  [ **CCL2** ]  2, MCP  [ **CCL2** ]  3, MCP  [ **CCL2** ]  4 on different processes linked to the chemokine receptor  **CCR5**  in recombinant CHO cells expressing the receptor at different levels.  Ref: 11861332 Br J Pharmacol, 2002
- In J774 cells, CpG ODN induced the chemokines MCP 1  [ **CCL2** ]   **CCL2**  and RANTES CCL5 and the chemokine receptors CCR1 and  **CCR5** .  Ref: 12538732 J Am Soc Nephrol, 2003
- In conclusion, variants of RANTES 28, MCP 1  [ **CCL2** ]  2518, and  **CCR5**  59029 neither influenced the incidence of acute rejection nor affected long term allograft survival upon liver transplantation in the context of this analysis.  Ref: 12462338 J Clin Immunol, 2002
- Recent studies have demonstrated that SMC possess a number of functional chemokine receptors, including  **CCR5** , CXCR4, and a receptor for monocyte chemoattractant protein 1  [ **CCL2** ]  MCP 1  [ **CCL2** ] .  Ref: 12851644 Microcirculation, 2003
- TAK 799 N, N dimethyl N 4 2 4 methylphenyl dihydro 5H benzocyclohepten 8 yl carbonyl amino benzyl tetrahydro 2H pyran 4 aminium chloride, a dual CCR2  **CCR5**  antagonist, demonstrated high affinity for the rhesus CCR2B in competition with 125I hMCP 1 binding to the receptor K i =0.5nM and also potently blocked the MCP 1  [ **CCL2** ]  induced calcium mobilization mediated through the receptor.  Ref: 12826275 Biochem Pharmacol, 2003
- At 26 weeks postirradiation, messages encoding the chemokines BLC now known as Scyb13, C10 now known as Scya6, IP 10 now known as Scyb10, MCP 1  [ **CCL2** ]  now known as Scya2  [ **CCL2** ] , MCP  [ **CCL2** ]  3 now known as Scya7, MIP 1gamma now known as Scya9, and RANTES now known as Scya5 and the chemokine receptors Ccr1, Ccr2,  **Ccr5**  and Ccr6 were elevated in fibrosis sensitive C57BL 6 mice.  Ref: 11839087 Radiat Res, 2002
- The PBMC levels of  **CCR5**  ligands MIP 1 alpha beta and RANTES, and chemokines MCP 1  [ **CCL2** ] , IL 8, lymphotactin, IP 10 and I 309 were analysed by ribonuclease protection assay RPA.  Ref: 12021948 J Neurol, 2002
- Whereas the amplitude of calcium mobilization induced by RANTES stimulation was lower in the  **CCR5**  CAM than the wild type WT receptor, MCP 1  [ **CCL2** ]  induced a higher signal in the CCR2 CAM than in CCR2 WT.  Ref: 12837756 J Biol Chem< MedlineTA>J Biol Chem, 2002
- In parallel, CCR1 and  **CCR5**  expression is down regulated and CXCL8,  **CCL2** , CCL3 and CCL5 production is up regulated.  Ref: 12548563 Eur J Immunol, 2003
- We investigated whether the functional polymorphisms of the chemokines RANTES, MCP 1  [ **CCL2** ]  and chemokine receptor  **CCR5**  are associated with the incidence of acute rejection and long term liver graft survival.  Ref: 12462338 J Clin Immunol, 2002
- In addition, no association of RANTES 28, MCP 1  [ **CCL2** ]  2518, or  **CCR5**  59029 variants with long term liver graft survival was found.  Ref: 12462338 J Clin Immunol, 2002
